# P17 Characteristics of pathogens and their susceptibility to the main groups of antibiotics in purulent-inflammatory diseases of soft tissues and post-operative purulent complications

**DOI:** 10.1093/jacamr/dlae136.021

**Published:** 2024-08-23

**Authors:** Oleksandr Viltsaniuk, Pavlo Belyaev

**Affiliations:** National Pirogov Memorial Medical University, Vinnytsia, Ukraine; National Pirogov Memorial Medical University, Vinnytsia, Ukraine

## Abstract

**Background:**

The problem of purulent-inflammatory diseases of soft tissues and post-operative purulent complications remains one of the most pressing problems of modern medicine. Despite the advances in the improvement of methods for fighting infectious complications, mortality in patients with suppurative and inflammatory processes remains high, which is primarily due to inadequate antimicrobial therapy and high antibiotic resistance of microorganisms. Therefore, the study of the nature of the microflora and its susceptibility to antimicrobial drugs remains an urgent problem.

**Objectives:**

To investigate the nature of pathogens and their susceptibility to the main groups of antibiotics in the patients of a general surgical hospital with purulent inflammatory diseases of soft tissues and post-operative purulent complications.

**Materials and methods:**

A retrospective review of the pathogens of purulent inflammatory processes of soft tissues and post-operative purulent complications, their susceptibility to the main groups of antibiotics in 704 patients who were treated in the surgical department for abscesses, phlegmon, post-operative wound suppuration and other purulent inflammatory processes were performed.

**Results:**

The analysis revealed that aerobic microflora was isolated in 560 patients (79.5%) and anaerobic microflora in 144 (20.5%). Among the isolated microflora, *Staphylococcus aureus* prevailed, which was isolated in 286 (40.6%) cases, *Staphylococcus epidermidis* in 115 (16.3%), *Escherichia coli* in 69 (9.8%) was in third place in terms of frequency of isolation, *Klebsiella pneumoniae* was isolated in 16 cases (2.3%), *Enterococcus faecalis* and *P. aeruginosa* in 26 (3.7%), and *P. vulgaris* in 6 (0.9%). *Candida* spp. were isolated in 15 (2.1%) patients and did not significantly affect the structure of pathogens of purulent inflammatory processes. Susceptibility testing of *S. aureus*, as the main causative agent of purulent inflammatory processes, showed its resistance to penicillin, aminoglycosides and macrolides. Third and fourth generation cephalosporins (63.1%), fluoroquinolones (76.3%) and lincosamides (70.8%) showed the highest activity against the isolated *Staphylococcus* strains.

**Conclusions:**

The main causes of purulent inflammatory diseases of soft tissues and post-operative purulent complications were *S. aureus* in 40.6% of cases, *S. epidermidis* in 16.3%, and intestinal bacteria. Third and fourth generation cephalosporins showed highest activity against the isolated *S. aureus* strains (63.1% of susceptible strains), followed by fluoroquinolones (76.3% of susceptible strains) and lincosamides (70.8%). Empirical therapy for purulent inflammation should take into account the microbial landscape of pathogens and their susceptibility to antibiotics in a particular general surgical hospital.

